# A Deep Learning Model for Preoperative Differentiation of Glioblastoma, Brain Metastasis, and Primary Central Nervous System Lymphoma: An External Validation Study

**DOI:** 10.3390/neurosci4010003

**Published:** 2022-12-31

**Authors:** Leonardo Tariciotti, Davide Ferlito, Valerio M. Caccavella, Andrea Di Cristofori, Giorgio Fiore, Luigi G. Remore, Martina Giordano, Giulia Remoli, Giulio Bertani, Stefano Borsa, Mauro Pluderi, Paolo Remida, Gianpaolo Basso, Carlo Giussani, Marco Locatelli, Giorgio Carrabba

**Affiliations:** 1 Fondazione IRCCS Cà Granda Ospedale Maggiore Policlinico, Unit of Neurosurgery, 20122 Milan, Italy; 2 Department of Oncology and Hemato-Oncology, University of Milan, 20122 Milan, Italy; 3 Unit of Neurosurgery, Ospedale San Gerardo, Azienda Socio-Sanitaria Territoriale di Monza, 20900 Monza, Italy; 4 School of Medicine and Surgery, University of Milano-Bicocca, 20900 Monza, Italy; 5 Unit of Neuroradiology, Ospedale San Gerardo, Azienda Socio-Sanitaria Territoriale di Monza, 20900 Monza, Italy; 6 Department of Pathophysiology and Transplantation, University of Milan, 20122 Milan, Italy

**Keywords:** brain metastases, deep learning, glioblastoma, machine learning, primary central nervous system lymphoma

## Abstract

**(1) Background:** Neuroimaging differentiation of glioblastoma, primary central nervous system lymphoma (PCNSL) and solitary brain metastasis (BM) represents a diagnostic and therapeutic challenge in neurosurgical practice, expanding the burden of care and exposing patients to additional risks related to further invasive procedures and treatment delays. In addition, atypical cases and overlapping features have not been entirely addressed by modern diagnostic research. The aim of this study was to validate a previously designed and internally validated ResNet101 deep learning model to differentiate glioblastomas, PCNSLs and BMs. **(2) Methods:** We enrolled 126 patients (glioblastoma: *n* = 64; PCNSL: *n* = 27; BM: *n* = 35) with preoperative T1Gd-MRI scans and histopathological confirmation. Each lesion was segmented, and all regions of interest were exported in a DICOM dataset. A pre-trained ResNet101 deep neural network model implemented in a previous work on 121 patients was externally validated on the current cohort to differentiate glioblastomas, PCNSLs and BMs on T1Gd-MRI scans. **(3) Results:** The model achieved optimal classification performance in distinguishing PCNSLs (AUC: 0.73; 95%CI: 0.62–0.85), glioblastomas (AUC: 0.78; 95%CI: 0.71–0.87) and moderate to low ability in differentiating BMs (AUC: 0.63; 95%CI: 0.52–0.76). The performance of expert neuro-radiologists on conventional plus advanced MR imaging, assessed by retrospectively reviewing the diagnostic reports of the selected cohort of patients, was found superior in accuracy for BMs (89.69%) and not inferior for PCNSL (82.90%) and glioblastomas (84.09%). **(4) Conclusions:** We investigated whether the previously published deep learning model was generalizable to an external population recruited at a different institution—this validation confirmed the consistency of the model and laid the groundwork for future clinical applications in brain tumour classification. This artificial intelligence-based model might represent a valuable educational resource and, if largely replicated on prospective data, help physicians differentiate glioblastomas, PCNSL and solitary BMs, especially in settings with limited resources.

## 1. Introduction

Preoperative classification of brain tumours represents a critical aspect of patient management. Brain metastases (BMs), glioblastoma and primary central nervous system lymphomas (PCNSLs) are among the most frequent intracranial neoplasms in adults (17%, 14.3% and 1.9%, respectively); hence, a correct diagnosis is a crucial point in the therapeutic path of a large number of patients worldwide [1,2,3].

In spite of the increased efficiency and popularity of MRI and the availability of advanced neuroimaging techniques that may assist in differentiating glioblastomas, BMs and PCNSLs, cases showing atypical features may prove challenging even for expert clinicians who spend a large proportion of their work time identifying, segmenting and classifying these lesions [4,5].

As far as the T1-weighted gadolinium-enhanced (T1Gd) images considered in this study are concerned, glioblastomas appear as iso-hypointense masses with necrotic-cystic areas and irregular contrast-enhanced margins similar to solitary BMs; however, atypical glioblastomas may show minimal or absent central necrosis.

PCNSLs, on the contrary, are usually shown on T1Gd images as iso-hypointense masses with a homogeneous enhancement within the entire lesion boundaries; in atypical presentations, there is central necrosis that may mimic glioblastomas [6], and the preoperative use of steroids in patients with PCNSLs may entail false negative pathological results, requiring additional invasive manoeuvres and potential harm and costs [7] to obtain the correct diagnosis.

In recent years, artificial intelligence (AI)—more specifically, deep learning (DNN)—has been accounted as an emerging and promising technique in supporting physicians in decision-making tasks based on MRI images (i.e., computer vision) [8,9,10,11,12].

The aim of this study was to develop a fast and reliable system for brain tumour classification in an experimental retrospective clinical scenario. In a previous investigation [13], we designed and internally validated a DNN model, achieving excellent diagnostic performance. The purpose of this study was the external validation of the model’s accuracy in differentiating GBMs, PCNSLs and BMs on T1Gd MRI scans and discussion of its eventual role in the amelioration of diagnostic and interventional workflows.

## 2. Methods

### 2.1. Study Definition

Ethical approval was waived by the two institutions involved, by the local Ethics Committees in view of the retrospective nature of the study and because all performed procedures were part of routine care. Informed consent was obtained from all participants included in the study. All procedures performed in studies involving human participants were in accordance with the Helsinki declaration.

An internal committee among authors (L.T., G.F., G.A.B., G.C., M.L.) was formed, and a consensus achieved on the current investigation’s proper design and reporting guidelines. An extensive review of “Enhancing the quality and transparency of health research” (EQUATOR) [14] network “https://www.equator-network.org” (accessed on 4 January 2022) contents was performed, and the “Standard for reporting of diagnostic accuracy study—Artificial Intelligence” (STARD-AI) [15] guidelines were selected and followed in the study protocol definition. The STARD-AI [15] guidelines were developed to report AI diagnostic test accuracy studies as an evolution of the previous STARD 2015 version [16], with the addition of a specific focus on designing and reporting evidence provided through AI-centred interventions. Adherence to STARD-AI recommendations was reviewed by the senior authors (G.C. and M.L.) throughout the investigation and during final review.

### 2.2. Patient Selection

The medical records and preoperative imaging of patients who underwent surgical tumour resection or biopsy at “Fondazione IRCCS Cà Granda Ospedale Maggiore Policlinico, Milan, Italy” (named Training Site or TrS) between June 2020 and April 2021 and at “Ospedale San Gerardo di Monza, Monza, Italy” (named Testing Site or TeS) between January 2018 and November 2021 were retrospectively collected. Patient data were included in the analysis if preoperative T1Gd MR images were available and histological analysis confirmed the diagnosis of glioblastoma, PCNSL or solitary BMs.

Patients were excluded if:

(1) Preoperative T1Gd MR images were absent or inadequate in quality, according to the senior neuroradiologists;

(2) They had previously received intracranial intervention (surgical intervention, gamma knife surgery or radiation therapy);

(3) Multiple enhancing lesions were detected on preoperative MRI;

(4) In glioblastoma cases, histopathological exams included testing for IDH mutations—hence, only IDH1 and IDH2 wild-type tumours were further considered in the investigation.

One-hundred twenty-one patients operated on at the TrS were selected to provide image data for the training dataset of our DNN model, as reported in a previous study [13].

A total of 126 patients met the inclusion criteria at the TeS and were selected for external validation of the aforementioned model.

### 2.3. MR Acquisition and Image Pre-Processing

The MR image scanning parameters at the TrS are reported elsewhere [13]. Concerning the MRI acquisition protocol at the TeS, all brain MRI studies were performed with a 1.5 T system (Philips^®^ Ingenia 1.5T CX), including axial T2-weighted imaging, fluid-attenuated inversion recovery (FLAIR) imaging, diffusion-weighted images (DWI) (a b-value of 1000 sec/mm^2^ and a single b-0 acquisition), susceptibility-weighted imaging (SWI), volumetric contrast-enhanced axial and sagittal T1Gd (Gadovist 1 mmol/mL; 0.1 mmol/kg body weight) imaging; ADC maps were calculated from isotropic DWI.

**Figure 1:**
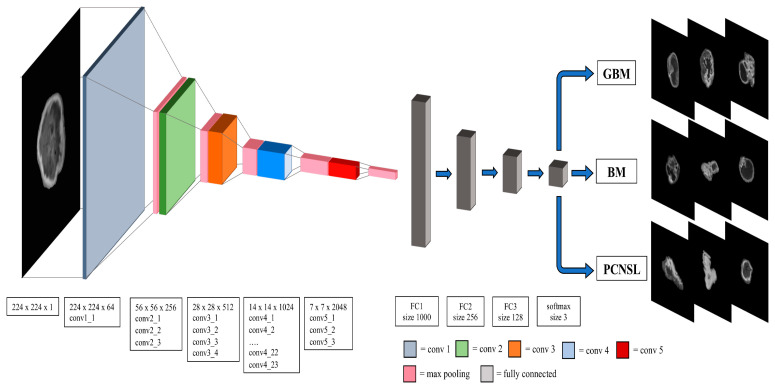
Model architecture trained as reported in Tariciotti et al. [13] and externally validated on the TeS dataset in the current study. The window size and stride for convolutional, maxpooling and fully connected layers are also presented. Conv: convolutional layer; FC: fully connected layer; GBM: glioblastoma; PCNSL: primary central nervous system lymphoma; BM: brain metastasis. “Reprinted with permission from Tariciotti et al. [13]. **Copyright** © 2022 Tariciotti, Caccavella, Fiore, Schisano, Carrabba, Borsa, Giordano, Palmisciano, Remoli, Remore, Pluderi, Caroli, Conte, Triulzi, Locatelli and Bertani. This is an open-access article distributed under the terms of the Creative Commons Attribution License (CC BY).

All MR images in the digital imaging and communications in medicine (DICOM) format were input to the Horos DICOM Viewer version 3.3.5, “www.horosproject.org” (accessed on 4 January 2022), a free, open-source medical imaging viewer and analytic tool. The lesions’ regions of interest (ROIs) were manually delineated on volumetric axial T1Gd scans. After segmentation and signal intensity normalization, all ROIs were then centred in a 224 × 224 pixels black box and exported in PNG file format (Figure 1).

### 2.4. Convolutional Neural Network Model

A 2D convolutional neural network model (i.e., ResNet-101) with 101 layers consisting of three-layer residual blocks pre-trained with the TrS dataset was used [13,17,18,19,20].

Each ROI was used as input for all three channels expected by the ResNet model and was treated as an independent image to increase the input data, though a group of slices was available for each patient. The predicted diagnostic class for each patient was the most frequently voted among its entire ROI set. The reported performance metrics were computed considering the number of correctly predicted patients and not the whole ROI dataset.

### 2.5. Performance Metrics

The classification performance of the DNN model was evaluated considering the following metrics:

(1) Area under the receiving operative characteristics curve (AUC-ROC):

(1) $$\mathrm{AUC}\;\left(f\right)=\frac{\sum_{t0\in D}0\sum_{t1\in D}1\left[f\left(t_{0}\right)<\;f\left(t_{1}\right)\;\right]}{\left|D^{0}\right|\cdot \left|D^{1}\right|}$$

where $1[f\left(t_{0}\right)<f\left(t_{1}\right)\;]$ denotes an indicator function, which returns 1 if $f\left(t_{0}\right)<f\left(t_{1}\right)$; otherwise, returns 0. $D^{0}$ is the set of negative examples and $D^{1}$ is the set of positive examples.

(2) Accuracy:

(2) $$\frac{TP+TN}{TP+TN+FP+FN}$$

where TP = true positive; TN = true negative; FP = false positive; FN = false negative.

(3) Precision or positive predictive value (PPV):

(3) $$\frac{TP}{TP+FP}$$

(4) Negative predictive value (NPV):

(4) $$\frac{\mathrm{TN}}{\mathrm{TN}+\mathrm{FN}}$$

(5) Recall or sensitivity:

(5) $$\frac{\mathrm{TP}}{\mathrm{TP}+\mathrm{FN}}$$

(6) Specificity:

(6) $$\frac{\mathrm{TN}}{\mathrm{TN}+\mathrm{FP}}$$

(7) F-1 score:

(7) $$2\times \frac{\mathrm{Precision}\times \mathrm{Recall}}{\mathrm{Precision}+\mathrm{Recall}}$$

A complete explanation of the parameters mentioned above is beyond the scope of the current study; further comprehensive descriptions are available elsewhere [21].

A one-vs-rest (OVR) multiclass strategy was employed to extract performance metrics for each outcome class. Then, the average value and its 95% bootstrap confidence interval were computed for each performance metric on the hold-out test set.

### 2.6. Human “Gold Standard” Performance

The tumour radiological assessment was addressed by experienced neuroradiologists (P.R. and G.B.) with at least 10 years of clinical experience. Electronic radiological reports were retrospectively reviewed to collect the primary radiological diagnosis. Afterwards, a comparison with the histopathological charts was completed, and the diagnostic classes were checked for discrepancies between radiological and pathological characterization. An OVR multiclass method was employed to extract neuroradiologists’ performance metrics for each outcome class.

### 2.7. Software and Hardware

All the statistical analyses were performed in a Jupyter Notebook using Python v.3.7.6 “https://www.python.org/” (accessed on 4 January 2022). The Python packages used for this study included: ‘PyTorch v1.7’ to develop and train the DNN model, ‘Numpy’ for Excel dataset handling; ‘Scikit-learn’ to compute performance metrics and ‘Seaborn’ to plot ROC-AUC. The workstation used to train the DNN model mounted an Intel Core i7–10700K processor, while the GPU was a Tesla K80 12GB.

## 3. Results

The cohort of selected patients included: 64 glioblastomas (mean age, 64.4 ± 9.04), 27 PCNSLs (mean age, 58.1 ± 16.5) and 33 BMs (mean age, 62.7 ± 14.2). A total of 2853 axial slices/ROIs of tumours were extracted, of which 1748 glioblastoma ROIs (mean ROIs 28.0 ± 19.0), 412 PCNSL ROIs (mean ROIs 15.0 ± 4.0) and 693 BMs ROIs (mean ROIs 21.0 ± 14.0). No significant differences in age, gender, number of total sequences or tumour ROI slice distributions were found between the three tumour groups (*p* > 0.05). The BM group included patients with various primary tumours, the most common of which being lung cancer (*n* = 16, 48.4% of all BMs), breast cancer (*n* = 5, 15.1%), gastrointestinal cancer (*n* = 4, 12.1%) and renal cancer (*n* = 3, 9.1%). Additional primary diagnoses were endometrial cancers and melanoma. Demographic characteristics are summarised in Table 1.

### 3.1. DNN Model Performance Metrics Evaluation

**Figure 2:**
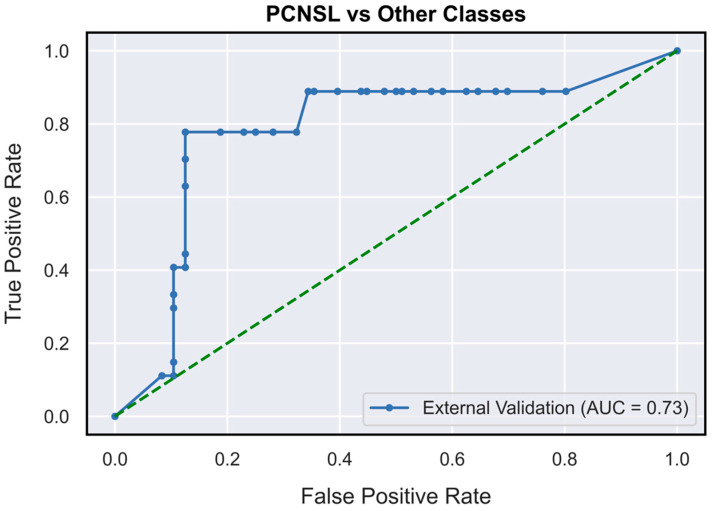
AUC-ROC curves (on TeS validation dataset) for PCNSL diagnostic outcome class (OVR). OVR: one-vs-rest; PCNSL: primary central nervous system lymphoma.

**Figure 3:**
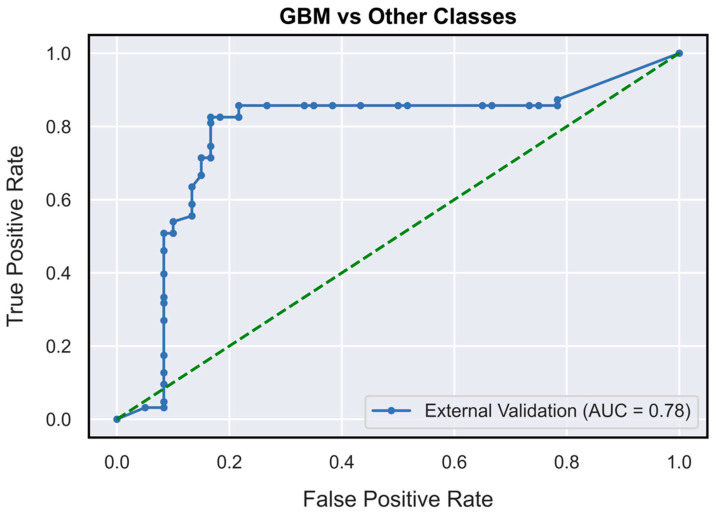
AUC-ROC curves (on TeS validation dataset) for glioblastoma diagnostic outcome class (OVR). GBM: glioblastoma; OVR: one-vs-rest.

**Figure 4:**
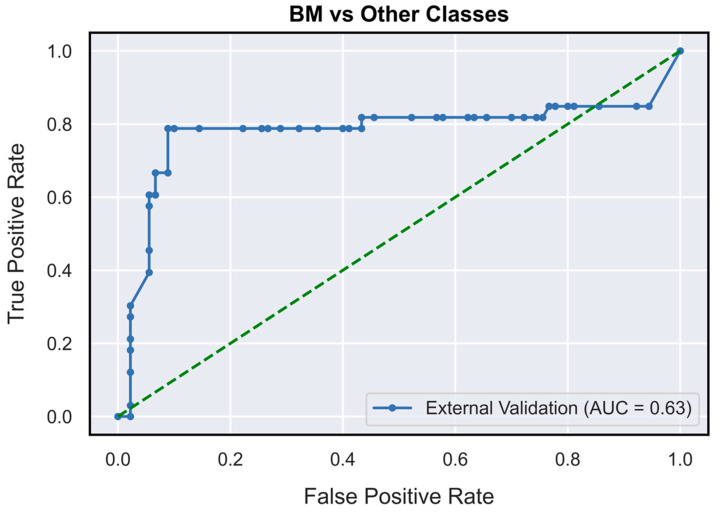
AUC-ROC curves (on TeS validation dataset) for solitary brain metastasis diagnostic outcome class (OVR). BM: brain metastasis; OVR: one-vs-rest.

**Figure 5:**
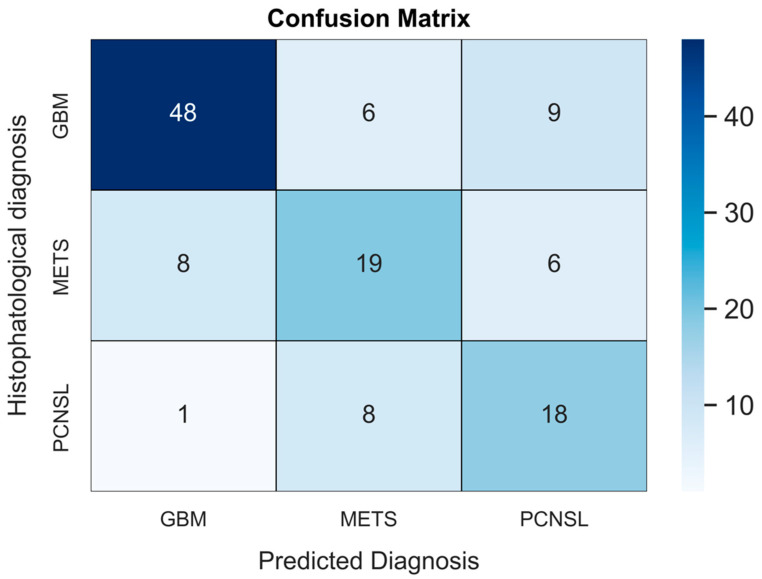
The confusion matrix (CM) shows the exact collocation of each patient among classification classes during a validated simulation with our DNN model. On the TeS patients’ data, the model misclassified histologically-confirmed atypical PCNSL nine times: in eight out of nine cases, the error led to a computer-based diagnosis of BM. On the contrary, among histologically-diagnosed BM, the model correctly identified 19 cases, while the remaining 14 cases were declared as glioblastomas (*n* = 8) and PCNSLs (*n* = 6). Overall, glioblastomas were more likely to be correctly diagnosed by the DNN model. The CM shows how the model chose among available diagnostic classes in the current work. BM: brain metastasis; CM: Confusion matrix; DNN: deep neural network; GBM: glioblastoma; PCNSL: primary central nervous system lymphoma.

The validated DNN model (Figure 1) achieved AUCs of 0.73 (95% CI: 0.62–0.85), 0.78 (95% CI: 0.71–0.87) and 0.63 (95% CI: 0.52–0.76), respectively, for the PCNSL (Figure 2), glioblastoma (Figure 3) and BM (Figure 4) diagnostic classes. High reliability was reported across all performance metrics for PCNSLs and glioblastomas diagnostic outcome classes, while lower reliability was reported for BMs. The complete performance metric evaluation and the related confusion matrix are reported in Table 2 and Figure 5.

### 3.2. Comparison of DNN Model and Neuroradiologists’ Gold Standard Performance

The performance metrics achieved by expert neuroradiologists are provided in Table 3. The DNN model showed a classification performance not inferior to the neuroradiologists’ gold standard reference on glioblastomas (F1 score 0.80 (0.73–0.87) vs. 0.81), PCNSL (F1 score 0.60 (0.50–0.73) vs. 0.59) and performed poorer than physicians in diagnosing BMs (0.57 (0.45–0.70) vs. 0.82).

## 4. Discussion

### 4.1. Performance Validation

In a previous study, we reported on a DNN model capable of efficiently and accurately differentiating glioblastomas, PCNSLs and BMs in an experimental “offline” environment [13]. Here, we externally validated the DNN model on “never seen” data gathered at an external academic site (TeS) with the comparable caseload, facility settings and technologies. The accuracy returned by our model was not inferior to a senior neuroradiologist’s performance in identifying PCNSLs and glioblastomas; accuracy for BMs identification was moderate, despite being lower than human evaluation.

In light of our previous preliminary findings, the evidence of model robustness and generalizability achieved in the current study supports the thesis of our DNN model being “experimentally not inferior” to senior physicians in classifying brain tumours in an unbiased cohort, endorsing the development and deployment of such models in medical training and clinical practice if cleared by regulatory authorities.

As previously documented, differentiating dubious BMs from gliomas and PCNSLs is challenging per se. Despite exponential advancements in the last decade, no single MRI modality can differentiate PCNSLs, BMs and glioblastomas with absolute accuracy. The search for a single sequence candidate to better classify these tumours has been limited to academic speculation, being restricted to synthetic scenarios rather than simulating clinical practice decision workflow, where multimodality is preferred. Indeed, results from previous studies are contradictory [22,23], with several authors reporting either T2-weighted, FLAIR or T1Gd scans’ superiority in brain tumour segmentation and classification [24,25,26]. The multimodality MRI approach recently showed promising diagnostic performance in differentiating brain neoplasms in experimental settings. Relevant findings were confirmed about dynamic susceptibility contrast (DSC) and apparent diffusion coefficient (ADC) maps combined with T1Gd-MRI scans. This multimodal approach came at the cost of an unstandardized diagnostic role due to the operator-dependent interpretation bias, high heterogeneity among brain tumour phenotypes and the additional need for hardware and set-up protocols, which might curb its use in facilities with limited resources [27,28,29].

During the study design, the authors agreed to implement T1Gd-MRI images only, relying on the greater worldwide availability of this sequence compared to diffusion and perfusion protocols, with the aim of extending the reproducibility of our workflow. Plus, the superior distinction of tumour borders and precise representation of central necrosis, which are common features of glioblastomas, atypical PCNSLs and BMs [30], facilitates manual segmentation avoiding ROIs’ drawing biases. However, the inclusion of additional sequences might have allowed a superior performance in the classification task.

Performance on BMs scored significantly lower compared to both the internal validation dataset and neuroradiologists’ performance metrics (accuracy: 77% vs. 81% vs. 89%, respectively [13]). This underperformance may be imputable to the great histological heterogeneity of this group of lesions and the consequent variability in radiological features. Additionally, a key distinguishing feature of BMs is abundant peritumoral oedema [31]; however, the peritumoural radiological environment was not included in the ROI segmentation of our dataset, which was limited to T1Gd boundaries. This might have influenced the lower performance of DNN on BMs, together with the neuroradiologists’ access to clinical history and additional imaging work-ups that the DNN model was blinded to. Indeed, while the model was blinded to any additional historical or diagnostic information except T1Gd scans, the diagnostic process accomplished at the time of imaging work-up comprehended additional characterization by means of total body CT, positron emission tomography (PET), and advanced MRI scans in a proportion of cases; being the retrospective evaluation of radiological reports set in routine clinical practice, we could not assess whether the aforementioned diagnostic exams—not involved in the current investigation—had a valuable impact on the putative radiological diagnosis. The comparative performance of DNN and senior neuroradiologists should be evaluated accordingly, and conclusions should be drawn carefully.

### 4.2. Perspective for Clinical Application and Public Health Impact

From a public health perspective, diagnostic tools such as our validated DNN model represent a promising technology spreading worldwide within industry, academia, and personal life settings. It is estimated that implementing AI algorithms in the USA might save USD 150 billion in healthcare costs by 2026 [32], with a net benefit even in lower-income countries, where AI experimentation is still under-practised. Implementation of AI protocols in healthcare is increasing in resource-poor countries of Asia and Africa collaterally to the wider availability of mobile phones, mobile health applications and cloud computing, which generate a sufficient mass of data to redirect to the purpose of studies like our own.

Given this, we believe that AI models might assist physicians in low-income countries in tackling macro and micro-scale healthcare disparities and might reduce healthcare borders and inequalities across high- and low-income countries by optimizing diagnostic workflows, augmenting physician performance in those settings where highly trained personnel are not routinely available or favouring teleconsultations and patient referral to more experienced hospitals. The whole process, as auspicated in high-income countries, might provide benefits to healthcare quality and allow weighted cost reduction [33], as suggested by a recent survey conducted in Pakistan [34]. However, our belief about the contributions of AI to healthcare optimization in such settings is speculative, and sufficient literature about AI use in resource-poor countries is still lacking to draw accurate previsions.

### 4.3. Perspective in Medical Education

Other than the previously discussed applications, efficiency of computer vision has already been demonstrated in other clinical scenarios (i.e., skin cancer classification, diagnosis of retinal disease, detection of mammographic lesions, fracture detection and many other tasks) [35,36,37,38].

Recent advancements have been made in integrating CV, and ML in general, into medical education and skill evaluation. Oliveira et al. reported a deep learning model called PRIME that is able to evaluate the microsurgical ability of different neurosurgeons in vessels dissection and micro-suture; the latter was designed with the aim of smoothing the microsurgical steep learning curve and providing a self-paced ML-advised tutor for continuous training without the need for any motion sensors around the operating table [39]. Similarly, Smith et al. reported a motion-tracking ML algorithm for surgical instrument monitoring during cataract surgery [40].

Finally, aimed to standardize surgical procedures, enhance training and lay the groundwork for future robot-assisted surgery, several groups are investigating whether DNN models can dissect surgical workflows into reproducible phases according to environmental exposure, segmentation of the anatomical scenario and instrument usage [41,42,43].

### 4.4. Strengths and Limitations

The DNN model hereby presented and validated on a cohort of more than one hundred patients is a simple but efficient tool able to help physicians diagnose atypical intracranial tumours with limited addition of human effort. Despite not being used in real-time scenarios yet, it is a promising and robust classification model and a candidate for further investigations in clinical trials. Nevertheless, several limitations restrict the generalizability of our results; the outcome accuracy was gauged in “offline” settings on a retrospective pool of image data. To date, the usefulness in actual clinical practice has been inferred but not demonstrated. In fact, while neuroradiologists with access to other relevant information scored as high as the DNN model in the majority of classes (and even higher on BMs), the interaction between the DNN response and the human decision-making process has not been experienced and evaluated. Further prospective trials are required to clarify the impact of artificial intelligence-based decision-making tools on human judgement and performance in clinical practice.

## 5. Conclusions

These results confirm the feasibility and reliability of our DNN model in experimental scenarios and open new possibilities for prospective clinical investigations. The delivery of such a diagnostic tool might enhance physicians’ performance and reduce the healthcare access gap in settings with limited human and instrumental resources. The validated model was built on an open-source programming language, and our methodology could be exported and further validated at different institutions.

## Figures and Tables

**Table 1:** Demographics and imaging acquisition data.

		Glioblastoma		BM		PCNSL		*p*-Value
		Count (N%)	Mean (SD)	Count (N%)	Mean (SD)	Count (N%)	Mean (SD)	
Gender	Female	26 (41.3%)		12 (36.4%)		8.0 (29.6%)		*p* > 0.05
	Male	37 (58.7%)		21 (63.6%)		19.0 (70.4%)		*p* > 0.05
Age (years)			64.4 (9.04)		62.7 (14.2)		58.5 (16.5)	*p* > 0.05
N° Slices of T1Gd sequence (N)			108.0 (52.0)		107.0 (59.0)		74.0 (61.0)	*p* > 0.05
N° Slices of ROI (N)			28.0 (19.0)		21.0 (4.0)		15.0 (14.0)	*p* > 0.05

Demographic characteristics of patients recruited at TeS. BM: brain metastasis; PCNSL: primary central nervous system lymphoma; ROI: region of interest.

**Table 2:** Performance metrics achieved by the convolutional neural network model in differentiating PCNSLs, glioblastomas and BMs.

Performance Metrics	PCNSL	Glioblastoma	BM
AUC	0.73 (0.62–0.85)	0.78 (0.71–0.87)	0.63 (0.52–0.76)
Accuracy	80.46% (74.8–87.01%)	80.37% (74.8–86.99%)	77.12% (71.54–83.74%)
Precision (PPV)	54.85% (44.11–70.00%)	84.13% (77.97–92.0%)	57.71% (46.67–72.73%)
Recall (Sensitivity)	66.86% (51.85–85.19%)	76.14% (66.67–85.71%)	57.04% (42.42–72.73%)
Specificity	84.29% (78.12–91.67%)	84.8% (78.33–93.33%)	84.49% (77.78–91.14%)
F1-Score	0.60 (0.50–0.73)	0.80 (0.73–0.87)	0.57 (0.45–0.70)

Performance metrics achieved on the hold-out test set were computed adopting an OVR multiclass strategy. Average value and 95% bootstrap confidence interval are reported. AUC: area under the curve; BM: brain metastasis; OVR: one-vs-rest; PCNSL: primary central nervous system lymphoma; PPV: positive predictive value.

**Table 3:** Neuroradiologist (Gold standard) performance metrics in differentiating PCNSL, glioblastoma and BM in the cohort examined.

Performance Metrics	PCNSL	Glioblastoma	BM
Accuracy	82.90%	84,09%	89.69%
Precision (PPV)	65.21%	87.50%	79.31%
Negative predictive value (NPV)	87.23%	81.57%	94.11%
Recall (Sensitivity)	55.55%	77.77%	85.18%
Specificity	91.11%	89.85%	91.42%
F1-Score	0,595	0,819	0,818

Performance metrics achieved by neuro-radiologists (defined as the gold standard) adopting an OVR multiclass strategy. The metrics were retrospectively computed by examining patient report charts: all patients underwent conventional plus advanced (T1-weighted, T2-weighted, FLAIR, diffusion-weighted, conventional T1-contrast-enhanced, dynamic contrast-enhanced and perfusion) MRI scans. Values were reported as single computation, so 95% bootstrap confidence intervals were not defined. BM: brain metastasis; OVR: one-vs-rest; PCNSL: primary central nervous system lymphoma; PPV: positive predictive value; NPV: negative predictive value.

## Data Availability

All authors confirm the appropriateness of all datasets and software used to support the conclusion. The dataset that supports the findings of this study is available from the corresponding author, L.T., upon request. The source code employed to develop the herein presented deep learning model is available from the corresponding author, L.T., upon request.
